# Cerebrovascular flow field reconstruction method based on residual network of physical information

**DOI:** 10.3389/fbioe.2026.1970931

**Published:** 2026-10-20

**Authors:** Zhifan Teng, Yaochun Hou, Jianhua Liu, Qiuliang Wang

**Affiliations:** 1 The School of Electrical and Information Engineering, Changsha University of Science and Technology, Changsha, China; 2 The State Key Laboratory of Disaster Prevention and Reduction for Power Grid, Changsha University of Science and Technology, Changsha, China; 3 State Key Laboratory of Industrial Control Technology, Zhejiang University, Hangzhou, China; 4 Huzhou Institute of Industrial Control Technology, Huzhou, China; 5 Institute of Electrical Engineering, Chinese Academy of Sciences, Beijing, China; 6 University of Chinese Academy of Sciences, Beijing, China

**Keywords:** cross-geometry generalization, hemodynamics, intracranial aneurysm, physics-informed neural network, surrogate modeling

## Abstract

**Introduction:**

Accurate characterization of the hemodynamic environment within intracranial aneurysms is important for rupture‐risk assessment and interventional treatment planning. Although computational fluid dynamics (CFD) provides high‐fidelity flow‐field data, its computational cost limits parametric analyses across multiple vascular geometries and inlet flow conditions. Existing physics‐informed neural‐network surrogates are predominantly trained for individual geometries and single flow conditions. Systematic comparisons of performance degradation under geometric and flow‐rate distribution shifts across different architectures remain lacking, and the distinct roles of physics‐based constraints under in-distribution versus out‐of‐distribution conditions have not been thoroughly examined.

**Methods:**

This study proposes PIReN, a physics‐informed residual network that decomposes the velocity‐pressure field into a learnable baseline field and a gated residual correction. The training objective incorporates continuity and steady, incompressible Navier‐Stokes momentum residuals. Training and in‐domain evaluation employ seven geometries; zero‐shot testing uses 16 fully held‐out geometries, and flow‐rate generalization is evaluated via five leave‐one‐flow‐rate‐out folds. These two protocols withhold complete geometries or flow conditions and avoid spatial-correlation leakage.

**Results:**

In the pointwise‐random in‐domain test, PIReN achieves an aggregate relative L_2_ error of 11.34% ± 0.65%, outperforming a standard PINN (16.57% ± 1.04%) and purely supervised MLP (14.74% ± 0.72%). These optimistic values describe spatial‐interpolation accuracy. Using a contiguous axial held‐out block while fixing model weights, PIReN still delivers superior accuracy: under the pooled configuration, its aggregate relative L_2_ error is 30.35%± 2.55% versus 39.27%±2.85% for PINN. PIReN dominates across all four metrics, both partitions and five configurations, with a larger absolute performance margin under the stricter block partition. On geometry set B, the corresponding errors for PIReN and the PINN are 42.71% and 121.92%, respectively.

**Discussion:**

Ablation studies show that removing the residual decoder produces the largest increase in prediction error, whereas the physics‐informed loss terms primarily reduce governing‐equation residuals. These results indicate that PIReN reduces performance degradation under changes in vascular geometry and inlet flow rate and provides a promising framework for steady‐state cerebrovascular flow‐field surrogate modeling.

## 1 Introduction

Intracranial aneurysms are common cerebrovascular lesions, and unruptured intracranial aneurysms affect approximately 3.2% of the general adult population (Vlak et al., 2011). Aneurysm rupture is associated with high mortality and disability rates (Brown and Broderick, 2014). Aneurysm formation, growth, and rupture depend not only on morphological and clinical factors but also on the local hemodynamic environment (Sforza et al., 2009). Patient-specific studies have identified systematic differences between ruptured and unruptured aneurysms in inflow concentration, flow complexity, and the spatial distribution of wall shear stress (Cebral et al., 2011; Xiang et al., 2011). Accurate characterization of the velocity and pressure fields within an aneurysm is fundamental to the analysis of local flow structures and hemodynamic changes before and after interventional treatment. In recent years, magnetically actuated soft microfiber robots (Liu et al., 2024) and fluid-driven magnetically controlled microcatheters (Wang et al., 2022) have shown promise for navigation through small-caliber vessels. Rapid flow-field estimation could provide real-time hemodynamic information to support the navigation and control of robotic systems during vascular interventions (Santiago-Dieppa and Friend, 2024).

Patient-specific computational fluid dynamics (CFD) enables vascular geometries to be reconstructed from medical images and provides flow variables, including velocity, pressure, and wall shear stress, that cannot be fully measured using clinical imaging modalities (Morris et al., 2016). However, a single CFD analysis requires image segmentation, geometry repair, mesh generation, and iterative numerical solution. When multiple vascular geometries or inlet flow conditions are considered, the resulting computational burden severely limits large-scale parametric studies and real-time applications (Arzani et al., 2021). To reduce this cost, various data-driven surrogate-modeling approaches have been developed, including the Fourier neural operator (FNO) (Li et al., 2021), Deep Operator Network (DeepONet) (Lu et al., 2021), graph neural networks (GNNs) (Suk et al., 2026), and surrogate models based on convolutional neural networks (CNNs) or transformer architectures. The Aneumo dataset was constructed from 427 patient-derived aneurysm geometries, and a DeepONet-SwinT benchmark was established for flow-field prediction (Li et al., 2025). Although purely data-driven surrogates can rapidly predict flow fields, they do not explicitly enforce mass and momentum conservation. Recent machine-learning benchmarks have also shown that complex geometries and unseen flow conditions can produce substantial prediction errors under out-of-distribution conditions (Rabeh et al., 2025; Karniadakis et al., 2021).

Physics-informed neural networks (PINNs) incorporate governing-equation residuals into the training objective and provide a means of inferring flow fields from limited observations (Karniadakis et al., 2021; Raissi et al., 2019). In cardiovascular and cerebrovascular applications, near-wall hemodynamics in stenosis and aneurysm models has been reconstructed using velocity measurements at only five sparse locations despite unknown inlet and outlet boundary conditions (Arzani et al., 2021). PINNs have been combined with transcranial Doppler ultrasound to reconstruct three-dimensional cerebral blood-flow velocity and pressure fields from sparse clinical measurements (Sarabian et al., 2022). An artificial-intelligence-based velocimetry method has enabled label-free three-dimensional flow-field measurements in a microaneurysm-on-a-chip system using only microscopy images (Cai et al., 2021). These studies demonstrate that governing-equation residuals can complement data-driven supervision in flow-reconstruction tasks with limited observations or boundary information. Subsequent work has extended physics-informed flow reconstruction to patient-specific aneurysm modeling: three-dimensional pressure distributions have been recovered from single-plane particle-image-velocimetry measurements in aneurysm models (Oldenburg et al., 2023), and transfer learning has been used to reduce the cost of repeated model training across patients (Daneker et al., 2024). Collectively, these studies illustrate the extension of physics-informed flow reconstruction from idealized numerical cases to patient-specific models, *in vitro* measurements, and medical-imaging-based applications.

Many existing PINN studies still train separate models for individual geometries or inlet conditions, primarily to solve a single forward or inverse flow problem rather than to construct a reusable parametric forward-surrogate model. Recent studies have begun to combine physical constraints with geometry encoding: vascular geometries have been represented using graph structures and evaluated on unseen carotid-artery geometries (Suk et al., 2026); point-cloud processing has been combined with physical constraints for cerebral blood-flow prediction across multiple cases (Liao et al., 2025); and neural solution frameworks have been compared for three-dimensional aneurysm flows (Moser et al., 2023). However, existing studies differ substantially in their data representations, training objectives, and evaluation protocols. In-domain accuracy, parameter efficiency, governing-equation residuals, and generalization to unseen geometries and inlet flow conditions have rarely been evaluated within a unified framework.

Cerebrovascular flow fields are jointly governed by vascular morphology, local geometric scales, and inlet flow rate, while velocity and pressure exhibit pronounced spatial coupling and nonuniformity (Ku, 1997). In a parametric surrogate that takes spatial coordinates and inlet conditions as inputs, a single network must represent both the global flow response and localized variations across operating conditions. With insufficient capacity, localized high-gradient structures may be difficult to resolve accurately; however, simply increasing network size does not necessarily produce consistent performance gains. Moreover, the supervised data loss, continuity residual, and momentum residual differ in numerical scale and optimization objective, potentially creating a trade-off between pointwise fitting accuracy and satisfaction of the governing equations (Wang et al., 2021).

To address the challenge of representing global flow responses and localized variations in a compact parametric cerebrovascular flow-field surrogate, this study proposes a physics-informed residual network termed PIReN. The model represents the velocity-pressure field through a baseline-field-residual decomposition and integrates baseline-field features with spatial and inlet-flow features using a dual-branch residual decoder and a channel-wise gating mechanism. Continuity and steady, incompressible Navier-Stokes momentum residuals are incorporated into the training objective to penalize violations of the governing equations. PIReN is systematically evaluated through in-domain reconstruction, parameter-efficiency analysis, architectural and loss-function ablations, cross-geometry testing, and leave-one-flow-rate-out testing. The evaluation considers reconstruction accuracy, governing-equation residuals, and performance degradation under changes in geometry and inlet flow rate.

The main contributions of this study are summarized as follows:

1. We propose a hierarchical velocity-pressure representation based on a baseline-field-residual decomposition and a dual-branch gated residual decoder that combines global flow responses with localized flow variations.
2. Through parameter-efficiency analysis and systematic ablation studies, we separately evaluate the effects of model capacity, architectural components, and physics-informed loss terms on flow-field reconstruction.
3. Within a unified training and evaluation framework, we assess the proposed model in three settings: in-domain spatial interpolation, cross-geometry generalization to unseen vascular geometries, and generalization to held-out inlet flow rates. Both reconstruction errors and governing-equation residuals are evaluated.

## 2 Methods

### 2.1 Governing equations

Given a spatial location within the vessel, the local equivalent diameter, and the mass flow rate, PIReN learns a parametric mapping from the conditioning variables to the corresponding three-dimensional velocity–pressure field:

$$s={\left(x,y,z,D,\dot{m}\right)}^{T}$$ (1)

where, x , y and z are the Cartesian coordinates, D denotes the local equivalent diameter of the cross-section at that location, and $\dot{m}$ is the mass flow rate. D > is evaluated pointwise on the vessel geometry, following the framework for geometric analysis of vascular structures as implemented in VMTK (Piccinelli et al., 2009). For each parametrically deformed model, the triangulated surface model provided by Aneumo is used as input. The Voronoi diagram of the surface is computed, and the centerline is obtained as the path of the centers of the maximal inscribed spheres through that diagram, by minimizing a cost function combining the local radius and the travelled distance. The model outputs the three velocity components and pressure at the specified spatial location:

$$q={\left(k,v,w,p\right)}^{T}$$ (2)

Where k , v and w are the predicted velocity components along the three orthogonal coordinate directions, and p is the predicted pressure. Under the assumptions of steady, incompressible Newtonian flow, the velocity and pressure fields satisfy the Navier–Stokes momentum equation and the continuity equation:

$$\rho \left(u\cdot \nabla \right)u=-\nabla p+\mu \nabla^{2}u$$ (3)

∇·u=0 (4)

where, $u={\left(k,v,w\right)}^{T}$ is the velocity vector and ∇ denotes differentiation with respect to spatial coordinates. And ρ and μ denote the fluid density and dynamic viscosity, respectively. Steady, incompressible Newtonian flow and rigid-wall assumptions are adopted. The fluid properties and boundary conditions are specified in the section on the dataset and experimental setup.

### 2.2 Network architecture

As shown in Figure 1, PIReN comprises a learnable baseline-field branch, a dual-branch residual decoder, and a gated fusion module followed by an output head. PIReN represents the predicted velocity–pressure field as the sum of a learnable baseline field and a residual correction:

$$\hat{q}\left(s\right)=q_{0}\left(s\right)+\Delta q_{\theta }\left(s\right)$$ (5)

where $q_{0}\left(s\right)$ denotes the learnable baseline field, as defined in Equation 2, and $\Delta q_{\theta }\left(s\right)$ denotes the residual correction produced by the residual decoder. The calculation of ***s*** refers to Equation 1.

**FIGURE 1:**
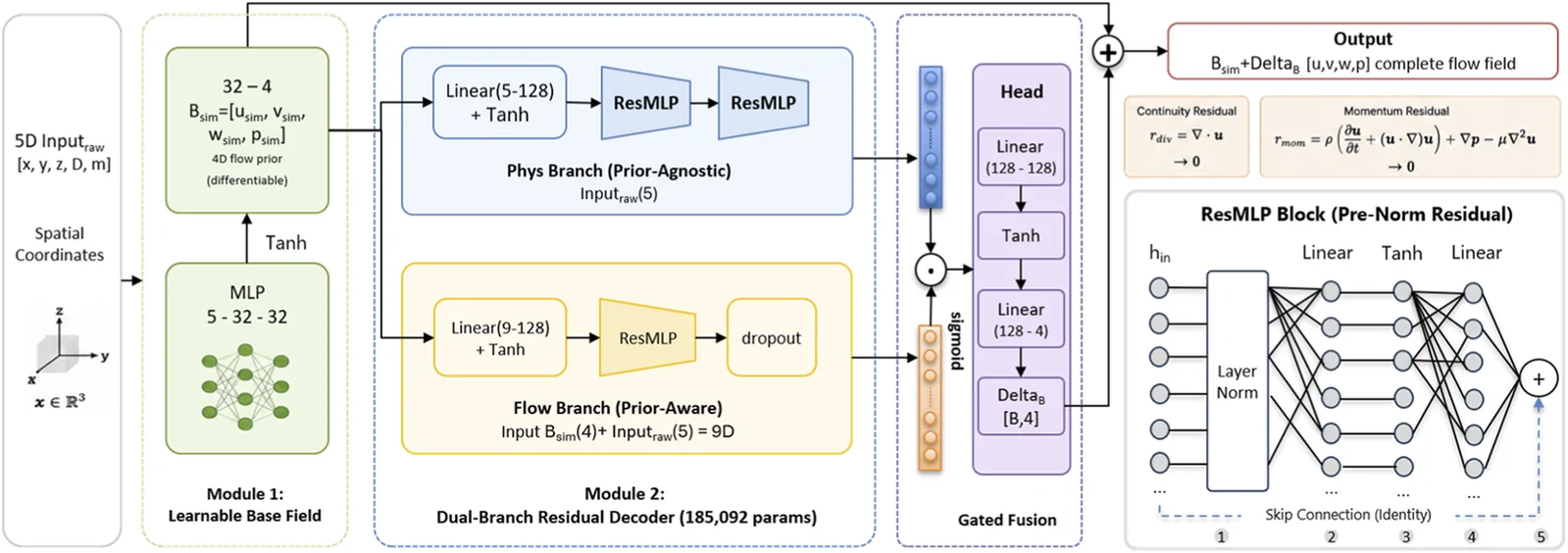
Architecture of PIReN. The baseline-field branch (left) maps a five-dimensional state vector to a four-dimensional baseline field. The dual-branch residual decoder (center) comprises a prior-agnostic physics branch and a prior-aware flow branch. Their features are fused through channel-wise gating and then mapped to a residual correction.

The baseline-field branch takes the five-dimensional state vector s as input, contains two fully connected hidden layers with 32 neurons each, and outputs the four-dimensional baseline field $q_{0}$ . Its forward mapping is given by

$$q_{0}\left(s\right)=W_{3}\sigma \left(W_{2}\sigma \left(W_{1}s+b_{1}\right)+b_{2}\right)+b_{3}$$ (6)

where $W_{1}$ , $W_{2}$ and $W_{3}$ are weight matrices, while $b_{1}$ , $b_{2}$ and $b_{3}$ are bias vectors. The function $\sigma \left(\cdot \right)$ denotes the nonlinear activation function. $\sigma \left(z\right)=\tanh\left(z\right)$ , and is used in every hidden layer of PIReN. The baseline-field branch learns a baseline flow-field representation conditioned on the spatial coordinates, local equivalent diameter, and inlet mass flow rate.

The residual decoder consists of two parallel subnetworks. The prior-agnostic physics branch takes the concatenated vector $\left[s;q_{0}\right]$ as input to extract residual features conditioned on the baseline field. The prior-aware flow branch takes only s as input and extracts conditioning features independently of the baseline-field output. Both branches have a hidden dimension of 128 and contain two stacked Pre-Norm residual multilayer perceptron blocks. Each block is defined as follows:

$$h_{pre}=\text{LayerNorm}\left(h_{\text{in}}\right)$$ (7)

$$h_{mid}=W\sigma \left(h_{\text{pre}}\right)+b$$ (8)

$$h_{\text{out}}=h_{\text{in}}+\text{Dropout}\left(h_{\text{mid}}\right)$$ (9)

where $\text{LayerNorm}\left(\cdot \right)$ denotes layer normalization. The Pre‐Norm design, as shown in Equation 7, normalizes the input features before nonlinear transformation, whereas the residual connection adds the transformed features (Equation 8) to the block input. Dropout is applied only in the prior‐agnostic physics branch for regularization, as described in Equation 9.

To combine the features extracted by the two branches, PIReN employs a channel-wise gating mechanism. The gating vector and fused feature representation are defined as:

$$g=sigmod\left(W_{g}\left[h_{f};h_{p}\right]+b_{g}\right)$$ (10)

$$h_{\text{fuse}}=g⊙h_{f}+\left(1-g\right)⊙h_{p}$$ (11)

where, $h_{f}$ and $h_{p}$ denote the outputs of the flow-field residual and prior-aware flow branch respectively; ⊙ denotes element-wise multiplication. The gating vector g is generated from the concatenated features of the two branches and adaptively weights their contributions on a channel-by-channel basis, as described in Equation 11.

The fused representation is passed through a two-layer output head to obtain the residual correction:

$$\Delta q_{\theta }\left(s\right)=W_{\text{ou}t2}\sigma \left(W_{\text{out}1}h_{\text{fuse}}+b_{\text{out}1}\right)+b_{\text{out}2}$$ (12)

The final flow-field prediction is obtained by combining the baseline field $q_{0}\left(s\right)$ with the residual correction $\Delta q_{\theta }\left(s\right)$ , as defined in Equation 5.

The nonlinear activation function sigma (.) is the hyperbolic tangent, $\sigma \left(z\right)=\tanh\left(z\right)$ , and is used in every hidden layer of PIReN: the baseline-field branch, both branches of the residual decoder, and the gating head. The gating vector of Equation 10 uses the logistic sigmoid, and the head output of Equation 12 is linear.

### 2.3 Physics-informed loss function

The PIReN training objective comprises a supervised data loss, a continuity-residual loss, and a momentum-residual loss:

$$L_{\text{total}}\left(e\right)=L_{\text{data}}+\lambda \left(e,c\right)L_{\text{cont}}+\lambda \left(e,m\right)L_{\text{mom}}$$ (13)

where, e denotes the training epoch, and $\lambda \left(e,c\right)$ and $\lambda \left(e,m\right)$ are the epoch-dependent weights assigned to the continuity- and momentum-residual losses, respectively. The supervised data loss is defined as the mean squared error between the predicted and reference flow variables:

$$L_{\text{data}}=\frac{1}{N_{\text{data}}}\sum_{i=1}^{N_{\text{data}}}{\left\|\hat{q}\left(s_{i}\right)-q_{\text{tr}\text{ue}}\left(s_{i}\right)\right\|}_{2}^{2}$$ (14)

The residuals are normalized using characteristic scales estimated on the training subset. Let $\sigma_{x}$ , $\sigma_{y}$ and $\sigma_{z}$ denote the standard deviations of the spatial coordinates over the training subset, and $\sigma_{k}$ , $\sigma_{v}$ and $\sigma_{w}$ those of the three velocity components. The characteristic length, velocity and pressure scales are defined as $L_{\text{char}}=\left(\sigma_{x}+\sigma_{y}+\sigma_{z}\right)/3$ , $U_{\text{char}}=\left(\sigma_{k}+\sigma_{v}+\sigma_{w}\right)/3$ and $P_{\text{char}}=\rho U_{\text{char}}^{2}$ , from which the continuity and momentum residual scales follow as $s_{\text{cont}}=U_{\text{char}}/L_{\text{char}}$ and $s_{\text{mom}}=P_{\text{char}}/L_{\text{char}}=U_{\text{char}}^{2}/L_{\text{char}}$ . We note that $U_{\text{char}}$ is a velocity-fluctuation scale and not a cross-sectional mean velocity, and that $L_{\text{char}}$ and $U_{\text{char}}$ depend on the training sample distribution as well as on the geometry.

Where, $N_{\text{data}}$ denotes the number of labeled data points. The continuity-residual loss penalises violations of incompressibility in the predicted velocity field. Its dimensionless form, as given in Equation 4, is defined as:

$$L_{\text{cont}}=\frac{1}{N_{\text{data}}}\sum_{i=1}^{N_{\text{data}}}{\left(\frac{\nabla \cdot u\left(s_{i}\right)}{s_{\text{cont}}}\right)}^{2}$$ (15)

Momentum residuals are used to constrain the steady‐state incompressible Navier‐Stokes equation construction. Referring to Equation 3, the loss is defined as follows:

$$L_{\text{mom}}=\frac{1}{N_{\text{data}}}\sum_{i=1}^{N_{\text{data}}}\frac{{\left\|\rho \left(\hat{u}\cdot \nabla \right)\hat{u}+\nabla \hat{p}-\mu \nabla^{2}\hat{u}\right\|}_{2}^{2}}{s_{\text{mom}}^{2}}$$ (16)

The operator ∇ denotes differentiation with respect to the spatial coordinates with D and $\dot{m}$ held constant. The derivatives are evaluated in physical units by applying the chain rule associated with the normalisation mappings, and are divided by $s_{\text{cont}}$ or $s_{\text{mom}}$ only afterwards. Because D enters as an independent input, automatic differentiation returns $dD/dx_{i}=0$ , and no term involving the spatial gradient of D appears in the residuals. The residuals consequently characterise the divergence and momentum balance of the field conditioned on a locally constant equivalent diameter. In regions where D varies rapidly, principally the bifurcations and the aneurysm neck, the divergence of the corresponding physical field defined on the vessel geometry contains an additional contribution proportional to ∇D , which is not represented here, so the reported DIV values are expected to underestimate the local mass-conservation error there.

First- and second-order spatial derivatives of velocity and the first-order spatial derivatives of pressure are computed using automatic differentiation. Because the model is trained with normalized coordinates and normalized output variables, the derivatives used in the governing-equation residuals are transformed back to physical units through the chain rule associated with the normalization mappings. The continuity and momentum residuals are nondimensionalised using the characteristic scales $s_{\text{cont}}$ and $s_{\text{mom}}$ defined above, so that the two physics-informed loss terms are of comparable magnitude before the weights $\lambda \left(e,c\right)$ and $\lambda \left(e,m\right)$ are applied.

To prevent the physics-informed loss from dominating the optimization during the early stages of training, a delayed cosine ramp-up schedule is used. For either physics-loss weight, denoted by $\lambda \left(e,\lambda_{\max}\right)$ , the schedule is defined as

$$\lambda \left(e,\lambda_{\max}\right)=\left\{\begin{array}{cc} 0, & e<e_{0}\\ \lambda_{\max}\cdot \frac{1-\cos\left[\pi \left(e-e_{0}\right)/T_{\text{ramp}}\right]}{2}, & e_{0}\leq e\leq e_{0}+T_{\text{ramp}}\\ \lambda_{\max}, & e>e_{0}+T_{\text{ramp}} \end{array}\right.$$ (17)

where, $\lambda_{\max}$ is the prescribed final weight, $e_{0}$ is the epoch at which the corresponding physics-informed loss is activated, and $T_{\text{ramp}}$ is the duration in epochs. Before epoch $e_{0}$ , training is driven solely by the supervised data loss. The physics-loss weight then increases smoothly to $\lambda_{\max}$ and remains constant after the ramp-up period.

## 3 Dataset construction and evaluation protocol

### 3.1 Dataset and preprocessing

A flow-field reconstruction dataset is constructed from the open-source Aneumo intracranial aneurysm CFD dataset [14]. For each sampled spatial point, the three-dimensional coordinates x , y , and z , local equivalent diameter D, and inlet mass flow rate m are used as model inputs, while the corresponding velocity components k , v , and w and pressure p are used as supervised targets. Five inlet mass flow rates are considered: $\dot{m}$ = 0.0025, 0.0030, 0.0035, 0.00375, and 0.0040 kg s^-1^, corresponding to Reynolds numbers of approximately 250–720. The CFD solutions serve as reference data for model training and evaluation. Blood density and dynamic viscosity are set to ρ = 1060 kg m^-3^ and μ = 3.45 × 10^−3^ Pa s, consistent with the values used in the original Aneumo simulations, and the boundary conditions follow those of that dataset.

Sampling and filtering proceed as follows. Points at which all three velocity components vanish are first removed, because they are no-slip wall points of the CFD mesh; this removes approximately 11%–13% of the points in each file. A fixed budget of 20,000 points is then drawn from each remaining file. Boundary conditions are not explicitly enforced in this pipeline. The loss function excludes boundary terms for inlets, outlets and walls, with both data-driven and physics-based losses evaluated only on sampled interior flow points after wall-point exclusion. The inlet and outlet flow conditions are inherited from the original Aneumo CFD reference data, and the rigid-wall no-slip assumption is implicitly satisfied via the governing equations and ground-truth fields, without additional boundary penalty terms.

Based on vascular morphology and spatial scale, the evaluation data are organized into two geometry sets. Geometry set A comprises seven parametrically deformed aneurysm models of one parent anatomy (Aneumo case IDs 18, 21, 26, 28, 32, 33, and 35), and geometry set B comprises sixteen parametrically deformed models of a second, distinct anatomy (case IDs 1–16); the two sets therefore originate from two different patient-derived anatomies rather than from a single common structure. Within each set, the deformation configurations form a graded sequence that varies aneurysm size and vessel caliber while preserving the parent anatomy. The two sets differ systematically in spatial scale (a centroid offset of approximately 3 cm between the two anatomies) and in morphological type (a small saccular aneurysm versus a large vessel-segment geometry), and their coordinate spaces do not overlap.

The local diameter is extracted with VMTK (version 1.5.2) from the STL surface models of Aneumo, using the centerline module; the seed points are taken at the inlet and at each outlet opening, of which each model has three. For geometry set A the resulting pointwise diameter D lies in the range 0.86–3.88 mm (median 3.19 mm), and for geometry set B in the range 0.37–4.46 mm (median 3.23 mm), the smaller values occur in the secondary branch distal to the bifurcation.

The CFD solutions under the various deformation configurations at different inlet mass flow-rate conditions constitute the supervised ground truth for training and testing. For the in-domain spatial-interpolation experiment, spatial points are sampled from each inlet-flow-rate condition in geometry set A using stratified random sampling and are partitioned into training, validation, and test subsets at a ratio of 8:1:1. This procedure preserves the proportion of samples from each flow-rate condition across the three subsets. All input and output normalisation parameters, together with the characteristic scales $s_{\text{cont}}$ and $s_{\text{mom}}$ used to nondimensionalise the governing-equation residuals (Section 2.3), are estimated exclusively from the training subset and then applied unchanged to the validation and test subsets.

Throughout this paper, three levels of description are used and are explicitly distinguished. (i) Geometry sets A and B denote the complete collections of 7 and 16 computational models; these sets define the population over which training and evaluation are performed. (ii) For qualitative illustration, Figure 2 displays one example model from each set; these panels serve only to illustrate vascular morphology and physical dimensions and support no quantitative claim. (iii) All quantitative results are aggregate statistics computed over the pooled evaluation point cloud as defined above; they are neither computed on nor restricted to the individual models displayed in Figure 2.

**FIGURE 2:**
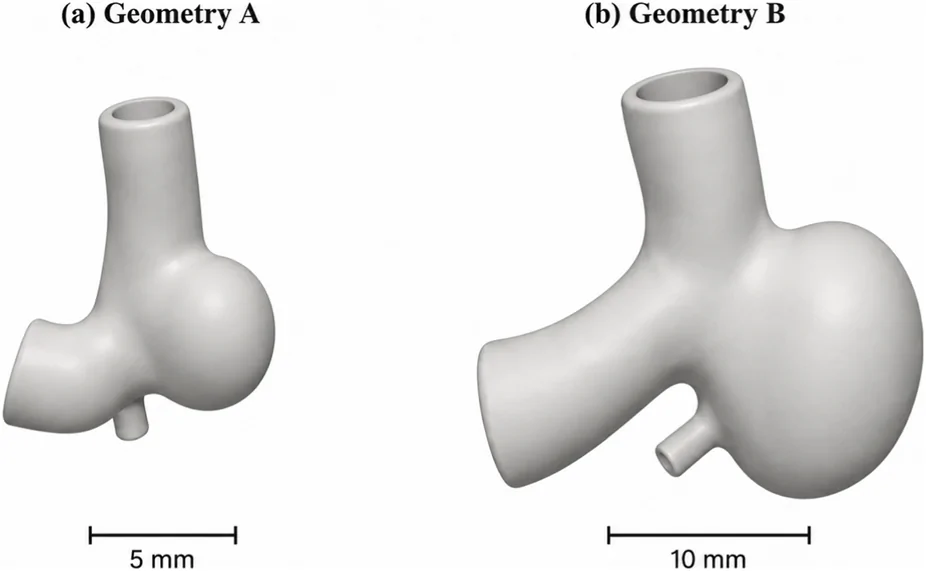
Morphology and dimensions of one example model from each geometry set. **(a)** Case ID18 from geometry set A; **(b)** case ID 12 from geometry set **(b)**.

### 3.2 Evaluation metrics

The models are evaluated from two perspectives: flow-field reconstruction accuracy and governing-equation consistency. The aggregate relative error $L_{2}$ and range-normalized mean absolute error (MNAE) quantify discrepancies between the predicted and CFD reference fields, whereas DIV and MOM measure violations of the continuity equation and the steady-state Navier–Stokes momentum equation, respectively.

For a scalar field q , the component-wise relative $L_{2}$ error is defined as:

$$L_{2}=\frac{{\left\|\hat{q}-q_{\text{true}}\right\|}_{2}}{{\left\|q_{\text{true}}\right\|}_{2}}$$ (18)

where, $\hat{q}$ and $q_{\text{true}}$ denote the predicted field and the CFD reference field, respectively. Equation 18 is evaluated both component-wise and jointly. For the component-wise errors, the predicted and reference fields of each output component (k, v, w, p) are denormalized from the network’s internal Z-score space back to physical units using the per-channel means and standard deviations estimated exclusively from the training subset. The relative L_2_ error is then computed separately for each component. For the aggregate (joint) relative error, the same de-normalization is applied, and the four de-normalized components are concatenated at each evaluation point into a four-dimensional vector; the per-point relative error is then

$L_{2,i}$ = ${\left\|\hat{q_{i}}-q_{i}\right\|}_{2}\;/\;{\left\|q_{i}\right\|}_{2}$ where $\hat{q_{i}}$ and $q_{i}$ are the predicted and reference vectors at the *i*th evaluation point, both in physical units. Because de-normalization restores each component to its physical scale, the aggregate per-point error is implicitly energy-weighted: components with larger physical magnitudes contribute proportionally more.

To quantify the mean pointwise prediction error, the MNAE error is defined as

$$\text{MNAE}=\frac{1}{N}\sum_{i=1}^{N}\frac{\left|{\hat{q}}_{i}-q_{\text{true},i}\right|}{q_{\max}-q_{\min}}$$ (19)

where, N is the number of sampled points in the test set, and $q_{\max}$ and $q_{\min}$ are the maximum and minimum values, respectively, of the corresponding CFD reference component over that test set. The aggregate MNAE is calculated as the unweighted macro-average of the MNAE values for the four output components.

To assess the satisfaction of the incompressibility condition by the predicted velocity field, DIV is defined as the dimensionless form of Equation 15 evaluated on the sampled test points, that is, the mean squared divergence residual:

$$\mathrm{DIV}=\frac{1}{N}\sum_{i=1}^{N}{\left(\frac{\nabla \cdot u_{i}}{s_{\text{cont}}}\right)}^{2}$$ (20)

where, ${\hat{u}}_{i}$ denotes the velocity at the *i*th evaluation point, whereas ∇ denotes differentiation with respect to spatial coordinates. DIV measures the local continuity residual at the sampled test points and should not be interpreted as the global inlet-to-outlet mass-flow imbalance.

To assess consistency with the steady-state Navier–Stokes momentum equation, MOM is defined as the dimensionless form of Equation 16 evaluated on the sampled test points, that is, the mean squared normalized momentum residual:

$$\text{MOM}=\frac{1}{N}\sum_{i=1}^{N}\frac{{\left\|\rho \left(\hat{u}\cdot \nabla \right)\hat{u}+\nabla \hat{p}-\mu \nabla^{2}\hat{u}\right\|}_{2}^{2}}{s_{\text{mom}}^{2}}$$ (21)

These two dimensionless metrics quantify the degree to which predicted flow fields satisfy the continuity and momentum governing equations. To facilitate quantitative comparison among different models, we introduce fixed reference thresholds for DIV and MOM to report the fraction of test-set points with residual values below these reference levels. Both thresholds apply to the dimensionless residuals defined above. $\mathrm{DIV}<0.01\Rightarrow \sqrt{0.01}=0.10$ , i.e., below 10% of the characteristic strain rate $s_{\text{cont}}=U_{\text{char}}/L_{\text{char}}$ , which for the present geometries is approximately $0.70\;s^{-1}$ ; $\text{MOM}<5\Rightarrow \sqrt{5}\approx 2.24$ times the characteristic inertial scale $s_{\text{mom}}=\rho U_{\text{char}}^{2}/L_{\text{char}}$ , approximately ${5.8\times 10}^{4}\;Pa\cdot m^{-1}$ . Neither threshold is derived from strict physical tolerance and neither acts as a hard pass-or-fail criterion in computation. They are pre-specified reference values applied uniformly across all competing models. MOM yields intrinsically larger values than DIV, as it accumulates residuals over three vector components and includes the comparatively noisy pressure-gradient term, which motivates its more permissive threshold.

### 3.3 Experimental design

PIReN is evaluated in four aspects: in-domain reconstruction, parameter efficiency, architectural and loss-function ablations, and generalization across geometries and flow rates. In addition, the sensitivity of the in-domain result to the data-partitioning scheme is assessed by the spatial-block cross-validation of Section 3.8. The in-domain reconstruction experiment compared PIReN with a standard PINN and a purely supervised MLP on previously observed geometries and inlet flow rates. The parameter-efficiency experiment compared model performance at different parameter counts by varying the hidden-layer width of the PINN and the hidden dimension of the PIReN residual decoder. The ablation studies separately assessed the contributions of the baseline field, residual decoder, gated-fusion mechanism, and physics-informed loss terms. Cross-geometry testing and five-fold leave-one-flow-rate-out testing evaluated generalization to unseen vascular geometries and held-out inlet flow rates, respectively. Except for their architectures and loss formulations, all models used identical data splits, normalization procedures, training budgets, and model-selection criteria. Models were trained with the Adam optimizer using an initial learning rate of 1 × 10^−3^ and a weight-decay coefficient of 1 × 10^−5^. The learning rate was adjusted according to validation loss using ReduceLROnPlateau, with a reduction factor of 0.5 and a minimum learning rate of 1 × 10^−6^. The batch size was 4096, and training was limited to 1,200 epochs. PIReN used a dropout rate of 0.05, and the final weights of the continuity- and momentum-residual losses were 0.1 and 0.001, In all configurations that include the corresponding residual loss, Equation 17 is instantiated as follows. For the continuity-residual loss, the weight $\lambda \left(e,c\right)$ of Equation 13 is obtained from Equation 17 with $e_{0}^{\left(c\right)}=15$ , $T_{\text{ramp}}^{\left(c\right)}=25$ and $\lambda_{\max}^{\left(c\right)}=0.1$ ; the weight is therefore zero before epoch 15, follows the half-cosine ramp of Equation 17 between epochs 15 and 40, and remains at 0.1 from epoch 40 onwards. For the momentum-residual loss, $\lambda \left(e,m\right)$ is obtained from the same equation with $e_{0}^{\left(m\right)}=40$ , $T_{\text{ramp}}^{\left(m\right)}=50$ and $\lambda_{\max}^{\left(m\right)}=0.001$ ; the weight is zero before epoch 40, ramps between epochs 40 and 90, and remains at 0.001 from epoch 90 onwards. The two ramps are sequential rather than simultaneous: the momentum term is activated at the epoch at which the continuity weight first attains its prescribed value, and the first 15 epochs constitute a warm-up period in which both weights vanish and training is driven solely by the supervised data loss of Equation 14. Both prescribed weights are attained well within the training budget of 1,200 epochs stated above, where the same values $\lambda_{\max}^{\left(c\right)}=0.1$ and $\lambda_{\max}^{\left(m\right)}=0.001$ are recorded. The ablation variants apply the same schedule to the residual terms they retain. Respectively. All experiments were conducted in PyTorch 2.7.0 with CUDA 11.8 on an NVIDIA A10 GPU. Three independent training runs are conducted using random seeds 42, 123, and 456 to verify reproducibility. For each trained model, the four per-point metrics—L_2_,ᵢ (Equation 18, evaluated per evaluation point as defined in Section 3.2), MNAE (Equation 19), DIV (Equation 20), and MOM (Equation 21) — are computed at every test point. Because each of the three seeds produces one prediction for each of the N = 14,000 test points, the three runs yield 3 N = 42,000 per-point values in total. Tables 1, 2 report the mean and standard deviation of these 42,000 values; the standard deviation therefore characterizes the spatial dispersion of the per-point errors and governing-equation residuals across the test cloud, not the variation across random seeds.

**TABLE 1:** Aggregate reconstruction errors and governing-equation residuals of the benchmark models.

Model	Parameters	$L_{2}$	MNAE	DIV	MOM
		Mean ± SD over 42,000 test-point predictions (3 seeds × 14,000 points)			
PIReN	186,472	11.34% ± 0.65%	1.11% ± 0.84%	0.0113 ± 0.0271	10.6099 ± 27.2089
PINN	199,940	16.57% ± 1.04%	1.61% ± 1.25%	0.0858 ± 0.1424	12.8160 ± 33.3983
MLP	173,956	14.74% ± 0.72%	1.84% ± 1.20%	4.7996 ± 9.8178	25.2491 ± 71.7847

**TABLE 2:** Comparison of parameter counts and flow-field reconstruction performance.

Model	Hidden dimension	Parameters	$L_{2}$	MNAE	DIV	MOM
			Mean ± SD over 42,000 test-point predictions (3 seeds × 14,000 points)			
PINN	64	13,124	24.13% ± 1.12%	2.50% ± 1.66%	0.1299 ± 0.2060	10.0024 ± 21.7268
PIReN	32	17,416	15.24% ± 0.91%	1.57% ± 1.09%	0.0356 ± 0.0696	12.6141 ± 38.1772
PINN	128	50,820	19.07% ± 1.08%	1.93% ± 1.39%	0.1119 ± 0.1923	10.5986 ± 25.7600
PIReN	64	53,288	13.25% ± 0.84%	1.33% ± 0.98%	0.0305 ± 0.0719	13.1374 ± 40.3878
PINN	256	199,940	16.57% ± 1.04%	1.61% ± 1.25%	0.0858 ± 0.1424	12.8160 ± 33.3983
PIReN	128	186,472	11.34% ± 0.65%	1.11% ± 0.84%	0.0113 ± 0.0271	10.6099 ± 27.2089
PINN	512	793,092	19.68% ± 1.25%	2.16% ± 1.93%	0.1675 ± 0.2858	10.6978 ± 16.1837
PIReN	256	698,600	13.26% ± 0.71%	1.49% ± 0.85%	0.0322 ± 0.0314	13.9329 ± 42.4217

### 3.4 Experiment 1: in-domain flow-field reconstruction

To evaluate spatial-interpolation performance for geometries and inlet flow rates represented in the training data, a purely supervised MLP, a standard PINN, and PIReN are compared on geometry set A in terms of their reconstruction of the three velocity components and pressure. Performance is evaluated using the aggregate relative $L_{2}$ error, MNAE, DIV, and MOM. A parameter-efficiency experiment is conducted to determine whether the performance gains of PIReN are attributable to increased model capacity. The network configurations of the standard PINN follow the architecture reported by Moser et al. [24], and the hidden feature dimensions of the PIReN residual decoder are set to 32, 64, 128, and 256. All configurations use the same training data and optimization protocol, and their training losses and validation metrics are tracked over the course of training. The parameter-efficiency analysis is restricted to the two physics-constrained models, namely, the standard PINN and PIReN. The purely supervised MLP serves only as a reconstruction baseline and is therefore excluded from the hidden-dimension parameter-efficiency analysis. This experiment is designed as a spatial-interpolation benchmark: it measures how accurately each model reconstructs the flow field at held-out spatial locations within geometries and inlet-flow-rate conditions that are represented in the training data. It is not a generalization test, and the errors it reports should not be compared with those of the block-level hold-out protocols of Sections 3.6, 3.7 and 3.8. Because all three models are trained and evaluated on identical partitions generated from the same seed, the identical split controls the partitioning condition across models; the spatial-block experiment is further used to assess whether the observed relative difference persists under reduced spatial proximity.

### 3.5 Experiment 2: ablation studies

To quantify the contributions of individual PIReN components to flow-field reconstruction performance, six ablation variants are derived from the full model, ${A1}_{\text{Full}}$ . (1) ${A2}_{\text{NoBase}}$ : The baseline-field branch is removed, and the residual decoder directly predicts the complete velocity–pressure field. (2) ${A3}_{\text{BaseOnly}}$ : The residual decoder is removed, leaving the baseline-field branch as the sole predictor of the velocity–pressure field. (3) ${A4}_{\text{NoGate}}$ : Channel-wise gating is replaced by feature concatenation followed by a linear projection for branch fusion. (4) ${A5}_{\text{NoDivNoMom}}$ : Both physics-based residual losses are removed, leaving only the supervised data loss. (5) ${A6}_{\text{DivOnlyNoMom}}$ : The continuity-residual loss is retained, whereas the momentum-residual loss is removed. (6) ${A7}_{\text{SemiSupervised}}$ : a supervision mask of $\left[1,0,0,1\right]$ is applied to the per-channel data loss, so that the streamwise velocity component u and the pressure p remain directly supervised while the two transverse components v and w are withheld from direct supervision and can be recovered only indirectly through the continuity and momentum residuals. The architecture, the data splits and the full physics loss, with $\lambda_{\text{cont}}=0.1$ and $\lambda_{\text{mom}}=0.001$ , are identical to ${A1}_{\text{Full}}$ , so the supervision mask is the only difference.

### 3.6 Experiment 3: out-of-domain cross-geometry generalization

An out-of-domain cross-geometry experiment is conducted to evaluate the model’s zero-shot generalization to unseen vascular geometries without parameter adaptation. Only data from geometry set A are used for model training, validation, hyperparameter selection, and checkpoint selection, whereas geometry set B remains strictly isolated from all of these procedures. After training, the spatial coordinates, local equivalent diameter, and inlet mass flow rate associated with geometry set B are provided directly to the trained model, with no additional training, parameter updates, or fine-tuning. Because geometry sets A and B differ in vascular morphology, spatial scale, and absolute-coordinate range, this experiment assesses zero-shot prediction under a compound distribution shift rather than generalization caused solely by morphological variation. Geometry B is evaluated at all five flow rates.

### 3.7 Experiment 4: in-domain inlet-flow-rate generalization

To evaluate generalization to inlet mass flow rates not observed during training for known vascular geometries, a five-fold leave-one-flow-rate-out experiment is conducted within geometry set A. In each fold, one inlet mass flow rate is completely withheld for testing, while data from the remaining four flow rates are used for training and validation. For each fold, the relative $L_{2}$ error, MNAE, DIV, and MOM are reported for both the held-out flow-rate condition and a reference set comprising flow rates observed during training. The difference between these results quantifies the degradation associated with generalization to an unseen inlet flow rate. When held out, the inlet mass flow rates of 0.0030, 0.0035 and 0.00375 kg/s assess conditional interpolation because they lie within the range spanned by the remaining training flow rates. In contrast, holding out 0.0025 and 0.0040 kg/s evaluates conditional extrapolation beyond the lower or upper boundary, respectively, of the flow-rate range represented in the corresponding training fold.

### 3.8 Experiment 5: spatial-block cross-validation

The in-domain experiment of Section 3.4 partitions the pooled point cloud of geometry set A at the level of individual spatial points. Because the velocity and pressure fields are spatially smooth, a test point can have near neighbours in the training subset, and the resulting errors characterise spatial-interpolation accuracy rather than generalization accuracy. To quantify this effect, the in-domain benchmark is repeated under a spatial-block cross-validation protocol in which the partitioning scheme is the only variable. The pooled cloud is divided into K = 5 contiguous blocks along the axial coordinate x by quantiles, so that each block spans approximately 20% of the streamwise extent of the domain. Leave-one-block-out is applied: in each fold, one block is held out in its entirety for testing, and the remaining K-1 blocks are split 80/10/10 into a training subset, a validation subset and a within-pool random test subset. Each of the five inlet-flow-rate conditions is processed separately, with 20,000 points per condition, so that is directly comparable with the pointwise-random-split baseline of Section 3.4. To ensure that the partitioning scheme is the only variable, the training budget and the random seed are matched to those of the baseline. Because a held-out axial block requires the model to reconstruct a contiguous vascular segment, including its streamwise pressure decay and any local recirculation, from a training pool that contains no point in that segment, this protocol is a region-wise extrapolation test and is strictly more demanding than the pointwise interpolation of Section 3.4. The two protocols are therefore reported as bracketing the attainable in-domain accuracy from the conservative and the optimistic side, rather than as one correct and one incorrect estimate.

## 4 Results and analysis

### 4.1 Experiment 1: in-domain flow-field reconstruction

Table 3 summarizes the component-wise relative L_2_ errors and parameter counts of the three benchmark models. PIReN contains 186,472 trainable parameters, comparable to 199,940 for the PINN and 173,956 for the MLP. With comparable model sizes, PIReN achieves an aggregate relative L_2_ error of 11.34% ± 0.65%, compared with 16.57% ± 1.04% for the PINN and 14.74% ± 0.72% for the MLP. These values correspond to error reductions of 31.6% and 23.1% relative to the PINN and MLP, respectively. Relative to the PINN, PIReN reduces the errors in k , v , w and pressure by 32.3%, 35.1%, 28.9%, and 27.1%, respectively. The corresponding reductions relative to the MLP are 22.5%, 25.6%, 24.2%, and 36.8%. Improvements are observed across all four output components, indicating that the reduction in aggregate error is not dominated by a single velocity component or by pressure.

**TABLE 3:** Comparison of component-wise relative errors $L_{2}$ and parameter counts for the three benchmark models.

Model	Parameters	$L_{2}$	$L_{2,k}$	$L_{2,v}$	$L_{2,w}$	$L_{2,p}$
PIReN	186,472	11.34% ± 0.65%	12.35% ± 0.93%	11.89% ± 0.69%	12.11% ± 0.89%	3.47% ± 0.66%
PINN	199,940	16.57% ± 1.04%	18.23% ± 1.22%	18.33% ± 1.41%	17.02% ± 1.44%	4.76% ± 0.99%
MLP	173,956	14.74% ± 0.72%	15.93% ± 1.12%	15.97% ± 0.94%	15.97% ± 0.84%	5.49% ± 1.43%

Table 1 further compares the aggregate reconstruction errors and governing-equation residuals. PIReN achieves an MNAE of 1.11%, lower than the values of 1.61% and 1.84% obtained by the PINN and MLP, respectively. The differences are more pronounced for DIV. PIReN yields a DIV of 0.0113, compared with 0.0858 for the PINN and 4.7996 for the MLP. PIReN also achieves the lowest MOM value of 10.6099, whereas the corresponding values for the PINN and MLP are 12.8160 and 25.2491. These results show that PIReN achieves both lower pointwise reconstruction errors and smaller violations of the continuity and momentum equations. Although the MLP attains a lower aggregate relative error $L_{2}$ than the PINN, its DIV and MOM values are substantially higher. Therefore, data-fitting accuracy does not necessarily imply physical consistency. A purely supervised model may recover the primary flow structures while still producing predictions that violate the governing equations. By contrast, PIReN provides a more favorable balance between numerical reconstruction accuracy and governing-equation consistency.

Figure 3 presents the spatial distributions of velocity magnitude, pressure, and governing-equation residuals on representative in-domain cross-sections. All three models recover the primary spatial structures of the CFD reference fields. The high-velocity regions, pressure gradients, and localized variations near vascular bifurcations are generally preserved, resulting in relatively limited visual differences among the predicted velocity and pressure fields. The governing-equation residuals reveal more pronounced differences among the models. For PIReN on the cross-sections shown in Figure 3, 72.1%% of the test points satisfy |DIV |<0.01, whereas the corresponding proportions for the PINN and MLP are 28.3% and 4.8%, respectively. Regions with high DIV are more localized in the PIReN predictions and are primarily concentrated in areas of complex local flow. This spatial pattern is consistent with the aggregate DIV results and indicates that PIReN produces lower continuity residuals at most test points. The spatial locations of large MOM values are similar across the three models. Approximately 89%–91% of the test points within the highest 10% of MOM values are located near vascular bifurcations. These regions are characterized by substantial changes in flow direction and large velocity gradients and are therefore more prone to elevated momentum residuals. Although PIReN does not substantially alter the locations of these high-residual regions, it reduces the overall MOM level. This result suggests that the spatial distribution of momentum residuals is strongly associated with local geometric and flow complexity.

**FIGURE 3:**
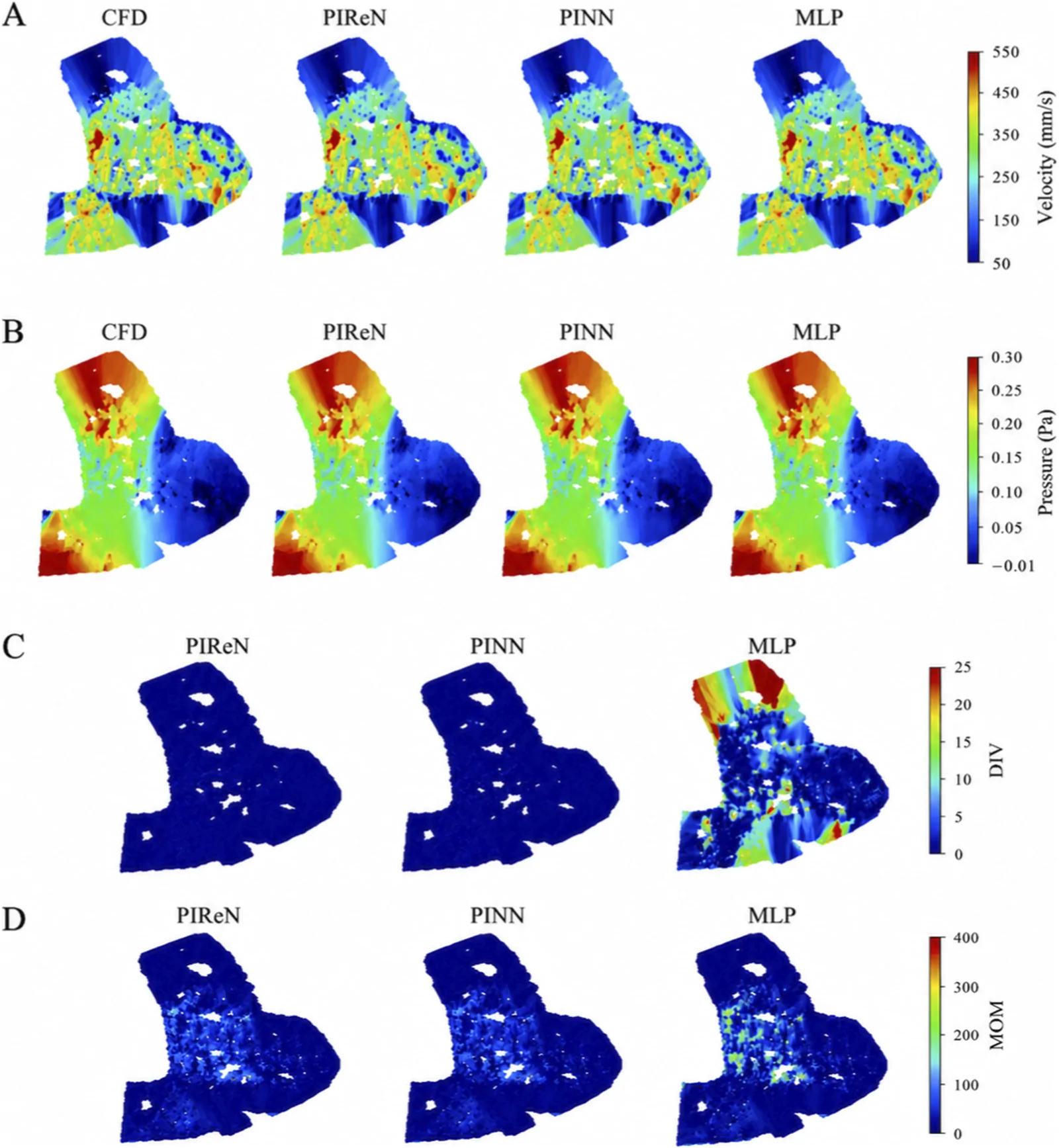
Spatial distributions of the CFD reference fields, model predictions, and governing-equation residuals for the three benchmark models: **(A)** velocity magnitude; **(B)** pressure; **(C)** DIV and **(D)** MOM. All panels show the same data-driven longitudinal section through the in-domain (Geometry A) test cloud: the plane passes through the centroid (28.13, 23.07, 28.94) mm with normal PC3 = (0.690, −0.714, 0.117), and points within ±0.455 mm of the plane (0.4σ_3_; slab thickness 0.91 mm) are retained, comprising 1,413 points.

Because the MLP exhibits substantially larger governing-equation residuals, the subsequent parameter-efficiency and generalization experiments focus on comparisons between PIReN and the PINN. Table 2 summarizes the performance of the two physics-constrained models at different parameter counts. As the hidden dimension increases, the aggregate relative $L_{2}$ error of the PINN decreases from 24.13% to 16.57% and then increases to 19.68%. Similarly, the error of PIReN decreases from 15.24% to 11.34% before increasing to 13.26%. Thus, neither model exhibits monotonic improvements in test performance as the parameter count increases. Across the four pairs of configurations with comparable parameter counts, PIReN consistently achieves lower aggregate relative error $L_{2}$ , MNAE, and DIV than the PINN. The performance advantage is most pronounced at moderate model sizes. PIReN-dim128 contains 186,472 trainable parameters and achieves an aggregate relative error $L_{2}$ of 11.34%, an MNAE of 1.11%, and a DIV of 0.0113. This configuration provides the lowest numerical errors and continuity residual among the evaluated PIReN variants. In contrast, MOM does not exhibit a consistent relationship with model size or a uniform advantage for either architecture. When the feature dimension of PIReN is increased from 128 to 256, the parameter count increases from 186,472 to 698,600, while the aggregate relative error $L_{2}$ increases from 11.34% to 13.26%. DIV and MOM also increase to 0.0322 and 13.9329, respectively. A similar degradation is observed when the PINN is enlarged to 793,092 parameters. These results indicate that increasing model capacity does not necessarily improve test performance. Excessively large models may increase optimization difficulty or lead to stronger fitting of the training samples. Considering reconstruction accuracy, governing-equation residuals, and parameter count, PIReN-dim128 is selected as the default configuration for the subsequent experiments.

Figure 4 illustrates the iterative convergence of PIReN-dim128. During the early stage of training, the $L_{2}$ error decreases rapidly from 63.25% at epoch 0%–15.98% at epoch 200, while MNAE decreases from 7.48% to 1.66%. Thereafter, both metrics improve more gradually and stabilize at approximately 11.5% and 1.13%, respectively, after about 600 epochs. DIV exhibits non-monotonic behavior during training. It initially increases from 0.1375 at epoch 0 to 0.3583 at epoch 20 and then gradually decreases to 0.0152 b y epoch 420. After approximately 600 epochs, DIV stabilizes at around 0.011. This trend indicates that the introduction of the physics-informed loss temporarily shifts the optimization balance, after which the numerical error and continuity residual gradually decrease and stabilize simultaneously.

**FIGURE 4:**
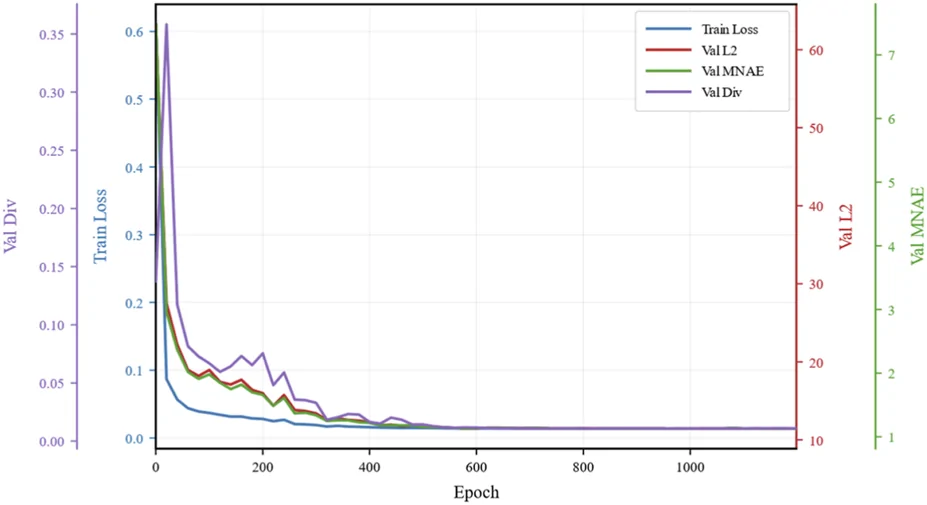
Validation error and continuity residual during training.

### 4.2 Experiment 2: ablation studies

Figures 5A,B compares the reconstruction errors and governing-equation residuals of the PIReN ablation variants. The results show that the residual decoder plays a critical role in flow-field reconstruction accuracy. After removal of the baseline field, ${A2}_{\text{NoBase}}$ yields an aggregate relative error $L_{2}$ of 12.16% and a DIV of 0.0442. When the channel-wise gating mechanism is replaced with simple concatenation, ${A4}_{\text{NoGate}}$ yields an aggregate relative error $L_{2}$ of 12.69% and a DIV of 0.0514. For both variants, the increase in the continuity residual is more pronounced than the increase in the reconstruction error, suggesting that removal of the baseline field or gating mechanism is associated with poorer reconstruction accuracy and weaker continuity consistency. The ${A3}_{\text{BaseOnly}}$ variant, which retains only the baseline field, exhibits substantial performance degradation. Its aggregate relative error $L_{2}$ and MNAE increase to 30.89% and 3.35%, respectively, while DIV rises to 1.2314. These results indicate that although the low-dimensional baseline field can represent the overall flow pattern, it cannot independently recover the complex flow structures near bifurcations and in regions with large velocity gradients. The residual decoder therefore provides the essential local corrections required for effective flow-field reconstruction.

**FIGURE 5:**
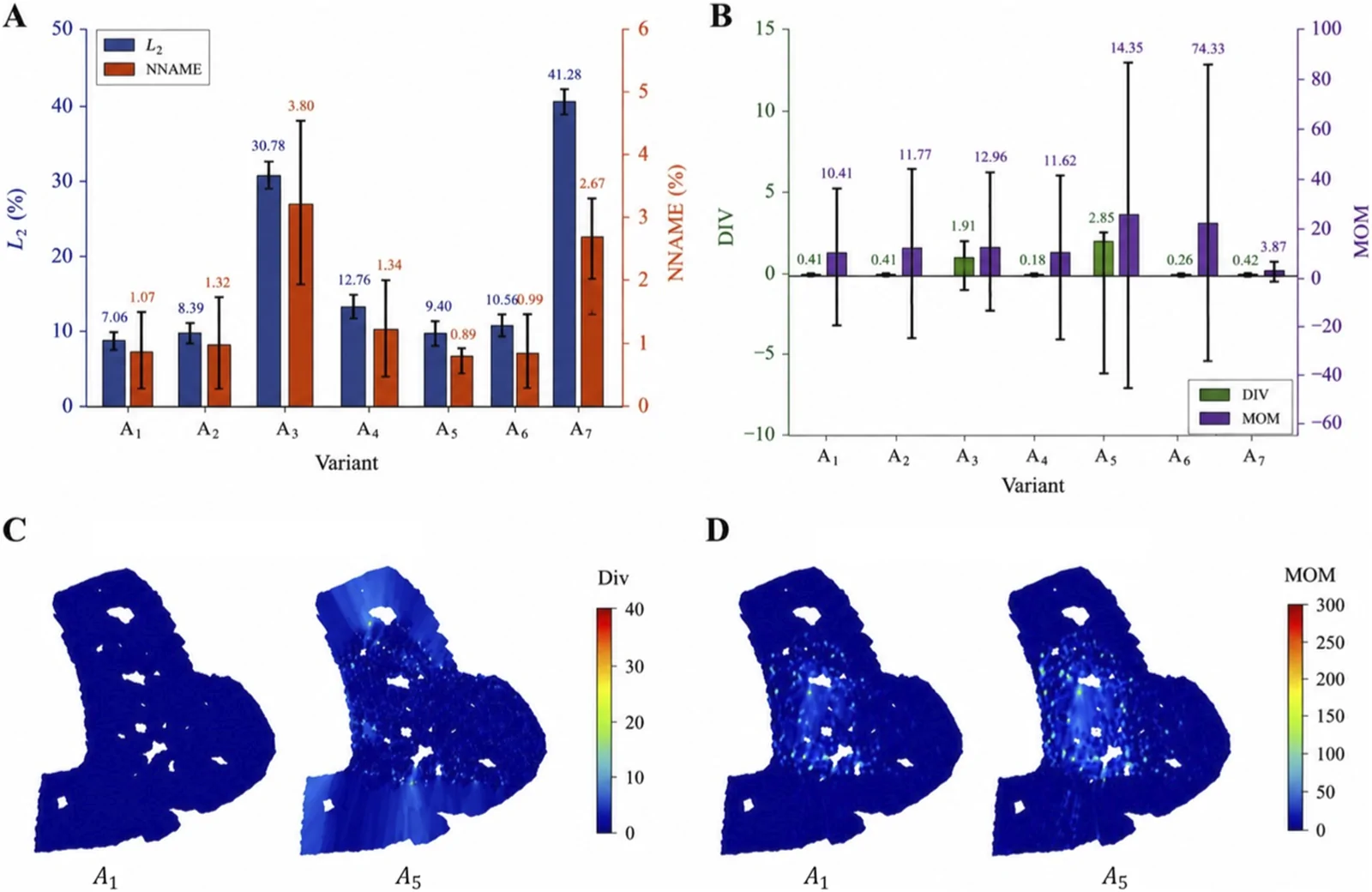
Comparison of **(A)** reconstruction errors and **(B)** governing-equation residuals among the PIReN ablation variants, and comparison of the spatial distributions of **(C)** DIV and **(D)** MOM between ${A1}_{\text{Full}}$ and ${A5}_{\text{NoDivNoMom}}$ **(C,D)** show the same longitudinal section through the Geometry A test cloud: the plane passes through the centroid (28.13, 23.07, 28.94) mm with normal PC3 = (0.690, −0.714, 0.117), and points within ±0.455 mm of the plane (0.4σ_3_; slab thickness 0.91 mm) are retained, comprising 1,413 points.

After both the continuity- and momentum-residual losses are removed, ${A5}_{\text{NoDivNoMom}}$ achieves a lower aggregate relative error $L_{2}$ of 9.64% and a lower MNAE of 0.88%. However, its DIV increases to 3.0351, approximately 269 times that of the full model, while MOM increases to 26.4607. Under the current supervised-data and loss-weighting configuration, these results indicate that the primary role of the physics-informed loss is not to further reduce the in-domain pointwise reconstruction error, but to suppress solutions that violate the governing equations. The ${A6}_{\text{DivOnlyNoMom}}$ variant, which retains the continuity-residual loss but removes the momentum-residual loss, yields a MOM of 21.6164. This value is substantially higher than that of the full model, although it remains lower than that of ${A5}_{\text{NoDivNoMom}}$ . This result indicates that continuity enforcement can reduce the divergence residual of the predicted velocity field but cannot replace explicit enforcement of the momentum equation. Under the incomplete-label setting, ${A7}_{\text{SemiSupervised}}$ maintains relatively low DIV and MOM values of 0.0160 and 4.2047, respectively, whereas its aggregate relative error $L_{2}$ increases to 41.39%. This contrast demonstrates that low governing-equation residuals alone do not guarantee accurate flow-field reconstruction.

Figures 5c,d further compares the local residual distributions of the full model, ${A1}_{\text{Full}}$ and ${A5}_{\text{NoDivNoMom}}$ . The DIV fields of the two models differ markedly. The residual field produced by the full model is dominated by low-value regions, whereas ${A5}_{\text{NoDivNoMom}}$ exhibits more extensive high-value regions along the main vessel segment and near bifurcations. For ${A1}_{\text{Full}}$ over all points of the in-domain test set, 43.2% of the test points satisfy |DIV|<0.01, compared with only 8.9% for ${A5}_{\text{NoDivNoMom}}$ . This result indicates that ${A5}_{\text{NoDivNoMom}}$ exhibits larger local continuity residuals at most sampled points and is therefore substantially less consistent with the incompressible mass-conservation condition than the full model. The spatial distributions of MOM are more similar between the two models. Nevertheless, a larger proportion of test points produced by ${A1}_{\text{Full}}$ satisfy the low-momentum-residual threshold. Specifically, 56.3% of the test points satisfy (MOM<5) for ${A1}_{\text{Full}}$ , compared with 42.8% for ${A5}_{\text{NoDivNoMom}}$ . These results suggest that jointly enforcing the continuity and momentum equations not only directly reduces the divergence residual but also improves momentum-equation consistency through physically informed regularization of the predicted velocity field.

### 4.3 Experiment 3: out-of-domain geometric generalization

Figures 6A–D presents the aggregate reconstruction performance of the two models on Geometry A and Geometry B. When evaluated on Geometry B, both models exhibit substantial increases in reconstruction error. The aggregate relative error $L_{2}$ of the PINN increases from 16.57% to 121.92%, corresponding to a 7.36-fold increase, whereas that of PIReN increases from 11.34% to 42.71%, corresponding to a 3.77-fold increase. Thus, PIReN exhibits substantially less performance degradation under the cross-geometry evaluation. On Geometry B, PIReN consistently outperforms the PINN across all four metrics. Compared with the PINN, PIReN reduces the aggregate relative error $L_{2}$ , MNAE, DIV, and MOM by 65.0%, 82.1%, 93.1%, and 82.2%, respectively. These results demonstrate that PIReN maintains lower reconstruction errors and governing-equation residuals on the unseen geometry.

**FIGURE 6:**
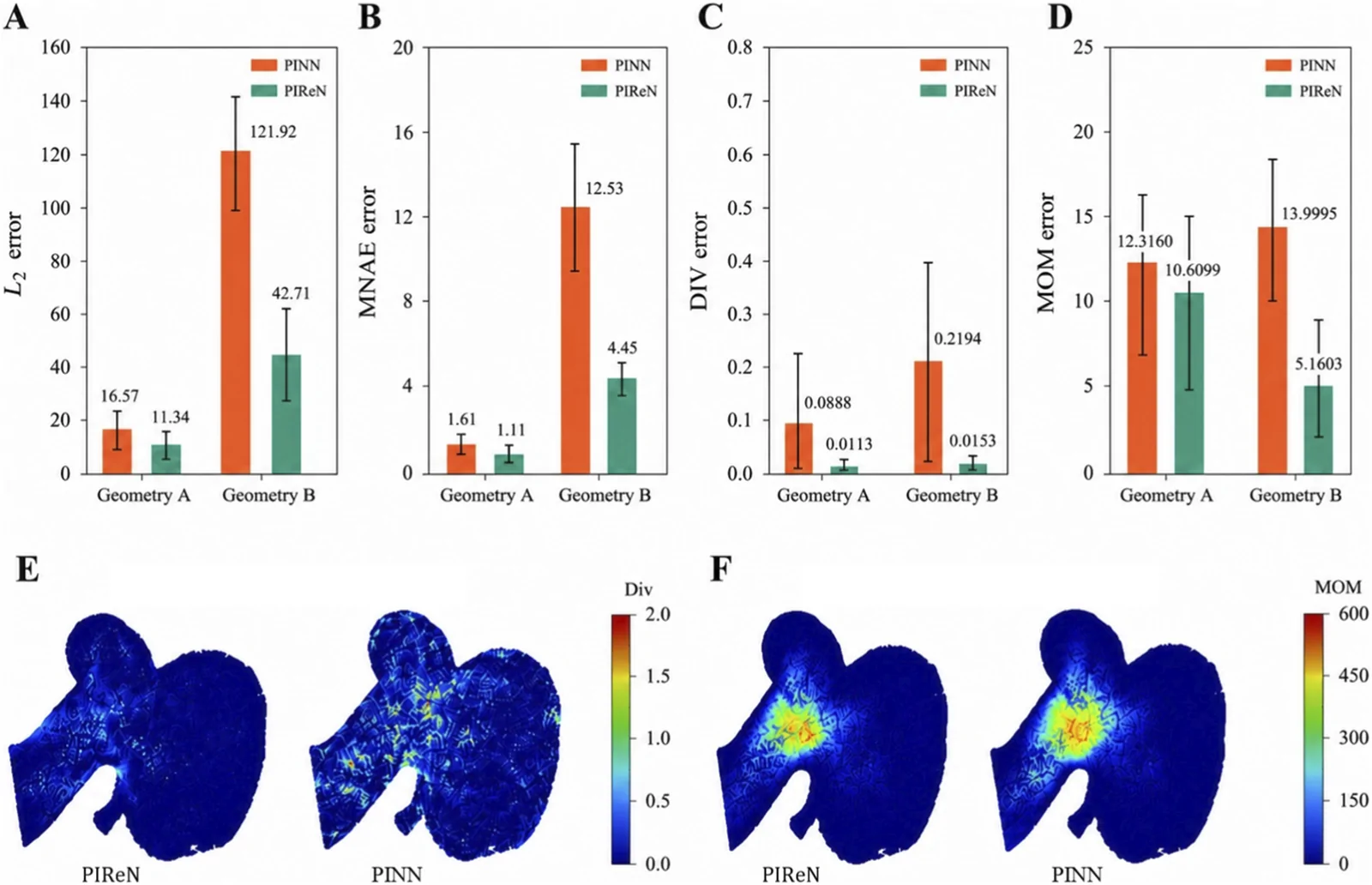
Comparison of **(A)** L_2_ **(B)** MNAE, **(C)** DIV, and **(D)** MOM errors between PINN and PIReN on Geometry A and Geometry B, and comparison of the spatial distributions of **(E)** DIV and **(F)** MOM on Geometry **(B,E,F)** show the same longitudinal section through the Geometry B (unseen) test cloud: the plane passes through the centroid (42.41, 46.35, 40.62) mm with normal PC3 = (−0.598, −0.769, +0.223), and points within ±0.628 mm of the plane (0.4σ_3_; slab thickness 1.26 mm) are retained (≈1.4 k points).

Figures 6E,F further presents the spatial distributions of the local residuals predicted by the two models on Geometry B. Large momentum residuals are primarily concentrated near vessel bifurcations and in regions with substantial curvature variations. Among the high-MOM points predicted by the PINN, 80.6% are located in bifurcation regions, compared with 68.5% for PIReN. These results indicate that regions with complex local geometry are major regions of elevated momentum residuals in cross-geometry prediction. PIReN also exhibits a smaller proportion of high-MOM points in these regions. The spatial distributions of DIV also differ markedly between the two models. The DIV field predicted by PIReN is dominated by low-value regions, with 47.4% of the test points satisfying |DIV|<0.01. In comparison, only 25.1% of the test points predicted by the PINN satisfy this threshold. These results indicate that PIReN better satisfies the local continuity condition on the unseen geometry.

It should be noted that the comparison above involves a compound distribution shift. The physical length scale of the two geometry sets is nearly identical: the characteristic diameter is 11.669 mm for Geometry A versus 11.531 mm for Geometry B (1.2% difference), and the bounding-box diagonals are 13.9 mm versus 13.4 mm. The compound shift is therefore dominated by two factors—morphology and coordinate frame—rather than by scale. The two centroids are 29.7 mm apart and the coordinate ranges are disjoint, while the PCA variance shares along PC1/PC2/PC3 change from 69.8/23.1/7.1% to 48.0/38.5/13.5%, i.e., from an elongated point cloud (σ_2_/σ_1_ = 0.58) to a nearly isotropic one (σ_2_/σ_1_ = 0.89). Because the current model represents geometry using absolute coordinates and local equivalent diameter, morphological variation and coordinate-frame extrapolation are coupled in this comparison and cannot be separated with the present experimental design. The reported degradation is therefore a system-level response to this compound shift, and the smaller degradation of PIReN is not attributed to any single factor.

### 4.4 Experiment 4: in-domain flow-rate generalization

Figures 7A–D presents the reconstruction results of the two models under the five held-out flow-rate conditions. PIReN consistently outperforms the PINN across all five conditions. The aggregate relative errors $L_{2}$ of PIReN under the held-out conditions range from 33.26% to 41.31%, whereas those of the PINN range from 71.12% to 152.22%. This substantial difference indicates that PIReN maintains more stable reconstruction performance when evaluated at flow rates excluded from training. PIReN also achieves lower MNAE, DIV, and MOM than the PINN under all five held-out conditions. Its advantage therefore extends beyond pointwise reconstruction accuracy to governing-equation consistency. The generalization difficulty nevertheless varies with the held-out flow rate. For PIReN, the largest aggregate relative errors $L_{2}$ occur when 0.0030 and 0.0035 kg/s are held out, whereas its errors are lower at 0.00375 and 0.0040 kg/s. For the PINN, the largest error occurs at 0.0025 kg/s, followed by 0.0030 and 0.0040 kg/s. These results indicate that flow-rate generalization performance depends on the specific operating condition withheld from training, although the underlying cause of this variation requires further investigation.

**FIGURE 7:**
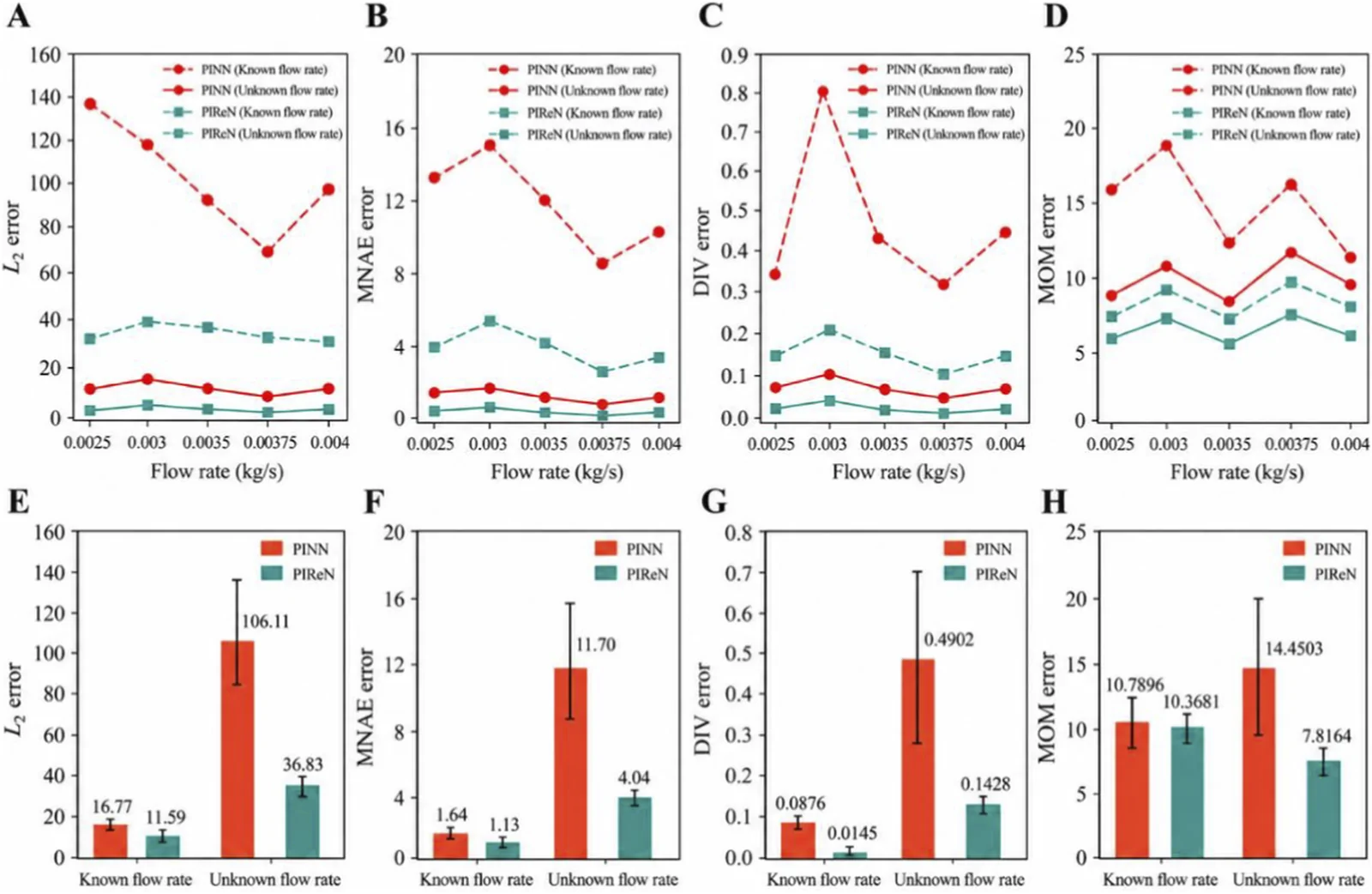
Comparison of reconstruction errors and governing-equation residuals between PINN and PIReN in the five-fold leave-one-flow-rate-out evaluation. Performance across different flow rates for **(A)** L_2_ **(B)** MNAE, **(C)** DIV, and **(D)** MOM. Average performance on the seen-flow reference and unseen-flow test sets for **(E)** L_2_ **(F)** MNAE, **(G)** DIV, and **(H)** MOM.

Figures 7E–H summarizes the average results of the five-fold leave-one-flow-rate-out evaluation. PIReN consistently outperforms the PINN under the held-out flow-rate conditions. Compared with PINN, PIReN reduces the average aggregate relative error from 106.11% to 36.83%, yielding a relative reduction of 65.3% calculated from the average errors. It also reduces the average MNAE from 11.70% to 4.04% and the average DIV from 0.4902 to 0.1428, corresponding to reductions of 65.4% and 70.8%, respectively. The average MOM is reduced from 14.1523 to 7.8546, corresponding to a reduction of 44.5%. PIReN also achieves lower aggregate relative error $L_{2}$ , MNAE, and DIV on the seen-flow reference sets. This indicates that its performance advantage is observed under both seen and held-out flow-rate conditions, particularly in terms of reconstruction accuracy and continuity enforcement. For both models, the reconstruction errors and DIV values under the held-out conditions are substantially higher than those on the seen-flow reference sets. These results show that changes in inlet flow rate remain a major source of performance degradation.

### 4.5 Experiment 5: Spatial-block cross-validation

Figures 8A–D provides the conservative counterpart to the in-domain figures. By holding out a contiguous axial block and evaluating with fixed model weights, this protocol removes any spatial correlation between test and training points. PIReN attains the lower error in every one of the forty pairwise comparisons formed by four metrics (L_2_, MNAE, DIV, MOM), two partitions (block held-out, within-pool), and five flow-rate configurations—forty out of forty, without exception.

**FIGURE 8:**
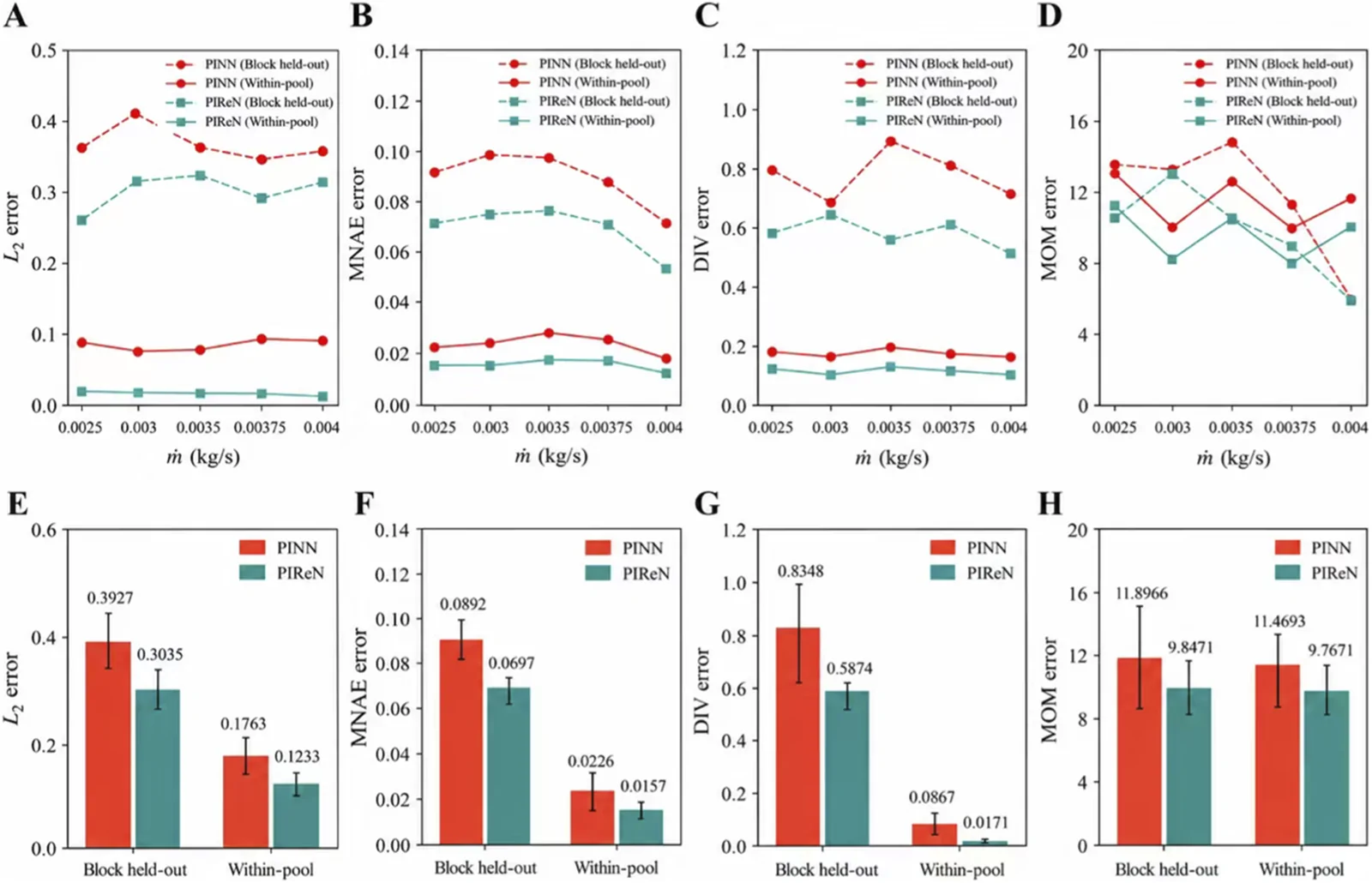
. Comparison of reconstruction errors and governing-equation residuals between PINN and PIReN in the five-fold Leave-one-block-out evaluation. Performance across different flow rates for **(A)** L_2_ **(B)** MNAE, **(C)** DIV, and **(D)** MOM. Average performance on the block check-out reference and within pool test sets for **(E)** L_2_ **(F)** MNAE, **(G)** DIV, and **(H)** MOM.

Averaged over the five flow-rate conditions Figures 8E–H, the aggregate relative L_2_ error is 0.3035 ± 0.0255 for PIReN against 0.3927 ± 0.0285 for the PINN under the held-out block, an absolute margin of 0.0892 and a mean relative reduction of 22.5% (range 13.2%–31.1%). The corresponding margins are 0.0196 for the mean normalized absolute error (reduction 22.0%, range 18.7%–25.0%), 0.2474 for the continuity residual (reduction 27.5%) and 2.0495 for the momentum residual (reduction 15.2%). Values are mean ± SD over the five flow-rate conditions (n = 5), each condition value itself being the mean over its five folds.

Two observations deserve emphasis. First, the absolute margin does not shrink when the partition is made stricter; it widens. The margin in aggregate relative L_2_ grows from 0.0530 under the within-pool random split to 0.0892 under the held-out block, that in mean normalized absolute error from 0.0069 to 0.0196, and that in the continuity residual from 0.0696 to 0.2474. Second, the relative reduction does narrow, from 30.1% to 22.5% in aggregate relative L_2_, but this is a denominator effect: both models become less accurate under the stricter partition, so the same absolute margin corresponds to a smaller percentage. The narrowing therefore reflects the shared degradation of both models, not a loss of standing of PIReN.

The advantage is also the more stable one. The cross-condition standard deviation of PIReN is smaller than that of the PINN for all four metrics under the held-out block—most markedly for the continuity residual (0.0518 against 0.1590, about one-third) — and for three of the four metrics under the within-pool split. PIReN is therefore not only more accurate on average but also more consistently accurate across operating conditions. Under the within-pool split the pool values of PIReN and the PINN (12.33% and 17.63%) lie within about one percentage point of the pointwise-random-split figures of Section 4.1 (11.34% and 16.57%), which confirms that the within-pool test set is a faithful proxy for that protocol.

## 5 Discussion

This study investigates flow-field representation and physics-based constraints in parametric surrogate modeling of cerebrovascular flow under varying geometric and operating conditions. In the in-domain experiments, PIReN, with 186,472 parameters, achieves lower reconstruction errors than both baseline models with comparable parameter counts. Specifically, the aggregate relative error L_2_ is 11.34% for PIReN, compared with 16.57% for the PINN with 199,940 parameters and 14.74% for the MLP with 173,956 parameters.

The ablations separate component roles: removing the residual decoder causes the largest accuracy loss (L_2_ 11.34% → 30.89%), whereas removing the baseline field or gated fusion raises DIV to 3.9–4.5 times that of the full model with only modest L_2_ and MNAE penalties. The physics loss is a trade-off, not an accuracy gain: removing the continuity- and momentum-residual terms lowers in-domain L_2_ to 9.64% but raises DIV from 0.0113 to 3.0351.

The two out-of-domain protocols probe different shifts. Leave-one-flow-rate-out holds the geometry fixed and tests interpolation between, and extrapolation beyond, the sampled inlet-flow-rate range; flow-rate extrapolation alone already raises L_2_ from 11.34% to 36.83%. The cross-geometry evaluation changes vascular morphology and absolute coordinate frame simultaneously while the physical length scale remains controlled (characteristic diameter 11.669 versus 11.531 mm, 1.2%); the two centroids are 29.7 mm apart with disjoint coordinate ranges, and the PCA variance shares change from 69.8/23.1/7.1% to 48.0/38.5/13.5% (σ_2_/σ_1_ = 0.58 versus 0.89). Because the model uses absolute coordinates and local equivalent diameter, morphological variation and coordinate-frame extrapolation are coupled and cannot yet be separated; the smaller degradation of PIReN (3.77-fold versus 7.36-fold for the PINN) is therefore a system-level comparison under a compound shift, not a controlled measurement of morphological generalisation.

Two quantitative features of Section 4.5 deserve emphasis. First, the absolute margin of PIReN over the PINN widens under the stricter partition: the L_2_ margin grows from 0.0530 (within-pool) to 0.0892 (block held-out), representing a 1.68-fold increase; the MNAE margin grows by a factor of 2.83; and the continuity-residual (DIV) margin by a factor of 3.56. The momentum-residual (MOM) margin grows by a factor of only 1.20 (from 1.70 to 2.05). Second, the relative reduction narrows: the L_2_ reduction decreases from 30.1% to 22.5%, and the DIV reduction from 80.3% to 27.5%. This narrowing is a denominator effect—both models degrade when tested on spatially disjoint data, so a larger absolute margin translates into a smaller percentage of a larger baseline error. The narrowing therefore reflects the shared degradation of the two models, not a change in their relative standing. The in-domain figures of Section 4.1 and the block-held-out figures of Section 4.5 thus bracket the attainable in-domain accuracy: the former as an optimistic bound (spatial interpolation) and the latter as a conservative bound (region-wise extrapolation along the vessel axis).

The generalization claims, grounded in Experiments 3 and 4 that withhold full geometries/flow conditions by design, remain unaffected. Future work should adopt a dimensionless coordinate parameterisation (x* = (x − c_g)/D_g, u* = u/U, p* = p/ρU^2^, with Re as an explicit input) to remove frame dependence, employ a graded out-of-domain protocol that varies morphology and coordinate frame one at a time, and evaluate the method on patient-specific geometries under unsteady flow conditions.

Extending PIReN to pulsatile flow requires temporal or phase information and inlet-waveform conditioning, because identical instantaneous flow rates can correspond to different dynamical states. The baseline and residual representations would need to capture this temporal dependence, and the momentum loss would include ρ∂u/∂t together with appropriate initial and time-varying boundary conditions. For compliant vessels, the decoder would additionally need to account for wall deformation and material properties. In an ALE or fluid–structure interaction framework, the flow prediction would depend on the moving computational domain, and kinematic and traction coupling would be required at the fluid–wall interface. These changes affect the conditioning, decoder, and loss formulation and have not been validated by the present steady rigid-wall experiments.

## 6 Conclusion

This paper proposes a physics-informed residual network, PIReN, for parametric cerebrovascular velocity–pressure field reconstruction. The model constructs a conditional flow-field mapping through a baseline-field–residual decomposition, a dual-branch residual decoder, and channel-wise gated fusion, and is trained with continuity and momentum-equation residuals as physics-based constraints. In-domain benchmark and parameter-efficiency experiments demonstrate that the performance improvement of PIReN is not solely attributable to increased parameter count. Ablation results show that removing the residual decoder causes the largest increase in numerical error, whereas the physics-based loss primarily reduces the governing-equation residuals and exhibits a trade-off with in-domain pointwise accuracy. In in-domain testing, PIReN achieves an aggregate L_2_ error of 11.34% (a 31.6% reduction relative to the PINN value of 16.57%); in the isolated cross-geometry test on geometry set B, the error is 42.71% (a 65.0% reduction relative to the PINN value of 121.92%); and the average error reduction across the five leave-one-flow-rate-out folds is approximately 65.3%. In the ablation experiments, removing the residual decoder increases the L_2_ error to 30.89%, whereas removing the physics-based loss reduces the L_2_ error to 9.64% but increases DIV to 3.0351. These results support the use of a baseline-field–residual decomposition for parametric cerebrovascular flow-field surrogate modeling. The in-domain figures are obtained under a pointwise random split and are bounded from the conservative side by the spatial-block cross-validation of Section 4.5; under that protocol the errors of both models increase while PIReN remains the more accurate of the two in every metric and configuration and its absolute margin over the PINN widens, cannot be explained solely by spatial correlation. The applicability of the method to independent patient-specific geometries and unsteady flow conditions requires further validation.

## Data Availability

The original contributions presented in the study are included in the article/supplementary material, further inquiries can be directed to the corresponding authors.
